# Supplementary material for: High Mobility Group Box 1 Is Potential Target Therapy for Inhibiting Metastasis and Enhancing Drug Sensitivity of Hepatocellular Carcinoma
Source: Int J Mol Sci. 2025 Apr 8;26(8):3491. doi: 10.3390/ijms26083491 (PMC12026555; doi:10.3390/ijms26083491)
Supplement: Supplementary file 1 [file ijms-26-03491-s001.zip › ijms-3476352-supplementary.pdf]

Supplementary Table S1. Correlation of nuclear/cytoplasmic HMGB1 expression and pathological parameters of patients with HCC.

Variables	Strongly positive nuclear HMGB1 expression, *n* (%)	*p*-value	Strongly positive cytoplasmic HMGB1 expression, *n* (%)	*p*-value
Gender				
Male	3 (9%)	0.3355	3 (9%)	0.6074
Female	1 (8%)		0 (0%)	
Age				
<60	2 (15%)	0.992	1 (8%)	0.4189
≥60	2 (6%)		2 (6%)	
T staging				
T1	3 (12%)	*0.0246**	2 (8%)	0.2693
T2	0 (0%)		1 (6%)	
T3	0 (0%)		0 (0%)	
T4	1 (20%)		0 (0%)	
M Staging				
M0	3 (7%)	0.4742	2 (5%)	0.5083
M1	1 (25%)		1 (25%)	
Tumor extension				
Confined to liver	3 (7%)	*0.0226**	3 (7%)	0.4541
Involves visceral peritoneum	1 (20%)		0 (0%)	
Vascular invasion				
Not identified	4 (13%)	0.4029	2 (7%)	0.255
Present	0 (0%)		1 (6%)	

Significant differences are indicated by *$p < 0.05$.
